# Common Causes of Failed Septoplasty: A Systematic Review

**DOI:** 10.7759/cureus.33073

**Published:** 2022-12-28

**Authors:** Khalid H Althobaiti, Abulkareem R Fida, Ahlam Almahmoudi, Dalal AlGhamdi, Manar Alharbi

**Affiliations:** 1 Department of Ear, Nose, and Throat, King Abdulaziz University Hospital, Jeddah, SAU

**Keywords:** septorhinoplasty, septoplasty, revision septorhinoplasty, revision septoplasty, nasal septum deviation, nasal septum, nasal obstruction, deviated septum

## Abstract

Failed septal correction is an undesirable outcome of primary septoplasty. In this systematic review, we aimed to assess all current studies concerning septoplasty failure, with a view to identifying its common causes. A systematic literature search was conducted by screening the PubMed, MEDLINE, Embase, and Cochrane Library databases for studies that assessed septoplasty failure and were published between January 2008 and January 2021. Three authors independently extracted information from each study and examined all included articles for bias. Four articles provided pertinent data regarding septoplasty failure. We gathered that missed nasal valve abnormality diagnosis, insufficient separation and resection of the bony-cartilaginous junction, and insufficient correction of caudal septal deviation could cause septoplasty failure. Additionally, iatrogenic problems, nasal asymmetry, and side-wall concavity involving the nasofrontal and columellar labial angles are contributing factors. Determining the cause of nasal blockage is challenging because it is subjective. Based on our findings, we concluded that in all patients with septal deviation, utmost care should be taken to avoid overlooking nasal valve abnormalities and other nasal diseases before conducting septoplasty. Moreover, inadequate correction of caudal septal deviation should be avoided. Furthermore, there is currently no widely accepted classification system for septal abnormalities to measure and describe septal deviation characteristics, making surgical planning and documentation difficult. Hence, further research that would lead to the creation of such a classification system is warranted.

## Introduction and background

The nose is an essential organ for survival and good quality of life in humans. It warms, humidifies, and cleanses the air entering the respiratory system, facilitating proper gas exchange in the lungs. Furthermore, its role as an olfactory organ enhances our ability to smell and taste food while protecting us from noxious stimuli. The intricate architecture and physiological complexity of the nose allow it to perform these critical functions [1].

One of the most common presenting complaints of patients consulting an otolaryngologist is nasal airway obstruction. Septal deviation is the most common cause of such obstructions. Studies of human skulls have revealed that septal deviation can be found in 75-80% of the population [2].

Septoplasty is a surgical procedure that is used to correct a deviated nasal septum (DNS). It should be distinguished from septorhinoplasty, a surgical procedure employed to manage the septum and other nasal structures [3]. In adults, surgical modification of DNS is the most common procedure performed by otolaryngologists [4]. Primary septoplasty has a success rate ranging from 43-85% [5], implying that at most 15% of septoplasty patients do not experience relief from symptoms. Several studies that have examined the causes of septoplasty failure have emphasized the significance of undetected nasal valve abnormalities. Incomplete or inappropriate correction of septum deformities also accounts for a considerable proportion of septoplasty failures. Other causes of persistent nasal obstruction after primary surgery include inappropriate management of turbinate hypertrophy and comorbid mucosal disease [5].

This systematic review aimed to identify the common causes of primary septoplasty failure and sought to compare the relative frequency of overlooked nasal pathologies with iatrogenesis associated with primary septoplasty.

## Review

Methods

Search Strategy

**Figure 1:**
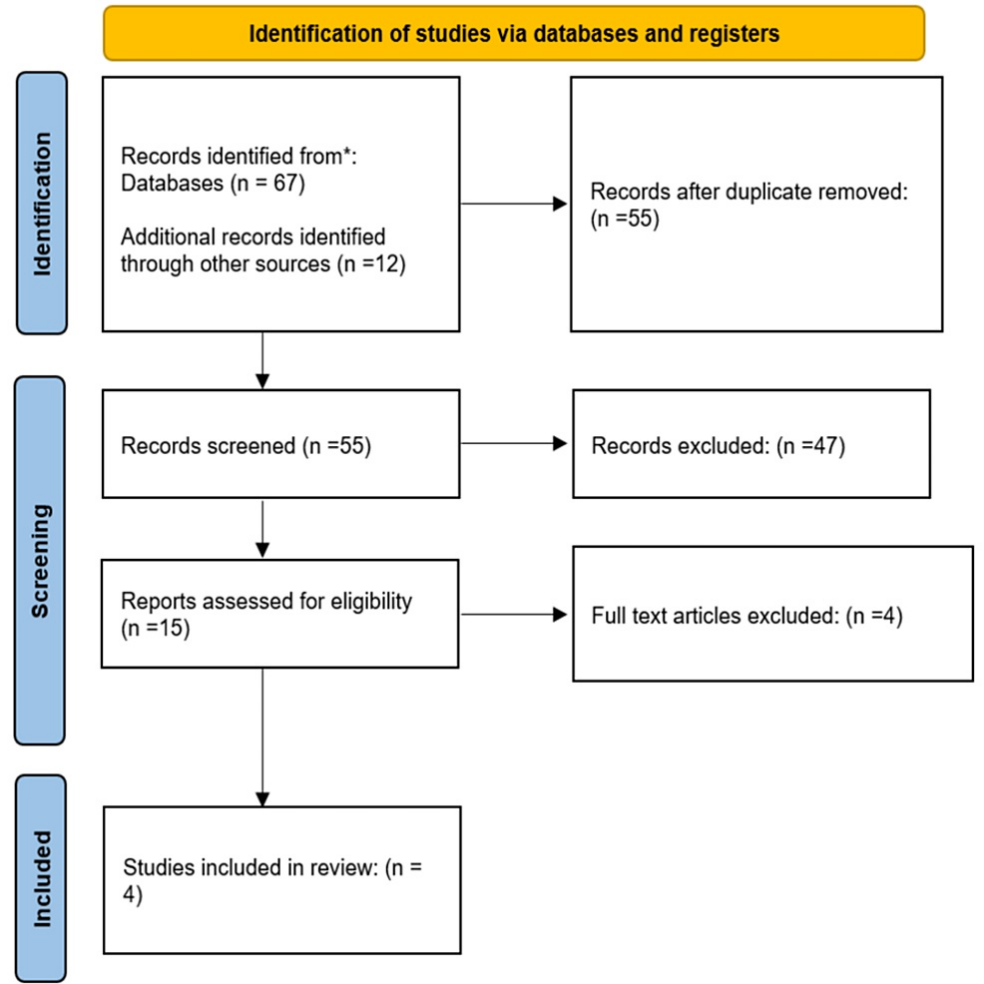
PRISMA diagram depicting the selection of articles PRISMA: Preferred Reporting Items for Systematic Reviews and Meta-Analyses

A systematic search was performed by screening the PubMed, MEDLINE, Embase, and Cochrane Library databases. These databases were searched for relevant papers published between January 2008 to January 2021 by using key terms linked to the nasal septum, nasal septum deviation, deviated septum, nasal obstruction, septoplasty, septorhinoplasty, revision septoplasty, and revision septorhinoplasty. We examined the abstracts of all identified articles that dealt with septoplasty failure and its causes. In addition, reference lists from the identified articles and those from recent review articles on this subject were reviewed. The Preferred Reporting Items for Systematic Reviews and Meta-Analyses (PRISMA) guidelines for reporting systematic reviews recommended by the Cochrane Collaboration were followed for performing this systematic review (Figure 1).

Inclusion Criteria

After consultation with an otolaryngologist, all studies published in English between January 2008 and January 2021 that addressed septoplasty failure and its causes were included. Eleven studies were eventually considered for this review.

Exclusion Criteria

All reviews, duplicate articles, studies that did not examine the causes of septoplasty failure, papers not published in English, and case reports/animal studies were excluded.

Data Extraction

Author names, year of study, sample size, study design, the timing of analysis, causes of septoplasty failure, results, and conclusions were all extracted by two investigators. Another investigator independently checked the extracted data for accuracy.

Assessment of Study Risk of Bias

The methodological quality of the selected articles was assessed by two investigators separately. The risk of bias was measured using pre-specified questions for each study design, and studies with a high risk of bias were excluded [6].

Results

Our review ultimately included four articles involving 260 patients. Three were retrospective studies, and one was a prospective study. All studies assessed the failure of septoplasty. A summary of the included studies is presented in Table 1.

**Table 1:** Summary of the reviewed studies

Author	Year	Study design	Sample size	Procedure	Results
Becker et al. [7]	2008	Retrospective	70	Patients who underwent revision septoplasty were identified, and information on their age at the time of the first operation and the period between the primary and revision surgeries was obtained	A significant number of patients who undergo revision septoplasty also have nasal valve collapse. We recommend that in addition to septal deviation and inferior turbinate hypertrophy, nasal valve function be thoroughly evaluated before performing septoplasty
Chambers et al. [8]	2015	Prospective	40	Patients with a history of septoplasty for nasal obstruction who underwent nasal valve repair with an open technique from January 1, 2012, to December 31, 2014, were included	Nasal valve dysfunction is still underrecognized, particularly in patients who have significant dorsal deflection and a narrow middle vault. The surgical repair of the nasal valve resulted in a substantial reduction in nasal blockage
Derin et al. [9]	2016	Retrospective	50	Patients with complaints of persistent or recurrent nasal obstruction after primary septoplasty and who underwent revision surgery between 2011 and 2015 were included	Iatrogenic abnormalities caused by surgery and diseases missed during the first septoplasty might result in persistent or recurrent nasal blockage
Jin et al. [10]	2018	Retrospective	100	Patients who had revision septoplasty due to persistent septal deviation from 2008 and 2014 were included	48 patients underwent revision septoplasty, and 52 underwent revision septoplasty combined with rhinoplasty. Nasal obstruction was the most common presenting symptom in almost all patients. Additionally, inadequate separation and resection of the bony-cartilaginous junction and insufficient restoration of the caudal septal deviation are the primary causes of protracted septal deviation following primary septoplasty

Becker et al. [7] performed a retrospective analysis of 70 patients who underwent revision septoplasty. They found a significant association between septoplasty failure and nasal valve collapse.

In contrast, Chambers et al. [8] published a prospective observational study performed in the United States that found a strong association between overlooked diagnosis of nasal valve collapse and septoplasty failure.

In 2016, Derin et al. [9] examined 50 patients who suffered from sustained or recurrent nasal blockage after initial septoplasty and underwent revision surgery from 2011 to 2015. The causes of sustained or recurring nasal blockage were determined to be diseases that remained undiagnosed during the initial septoplasty and iatrogenic problems brought on by the surgery.

Jin et al. [10] published a retrospective analysis of 100 patients who underwent revision septoplasty for chronic septal deviation between 2008 and 2014. Among these, 52 patients underwent revision septoplasty alongside rhinoplasty, and 48 underwent revision septoplasty alone. Their research revealed that nasal blockage was the most prevalent complaint among individuals.

Discussion

Septoplasty is among the most commonly performed surgical interventions for addressing nasal obstruction [11]. Patients are often concerned about complications associated with this surgery since the nose is the most noticeable facial feature [12]. This systematic review aimed to assess all current studies concerning septoplasty failure, in order to identify its common causes.

Four studies in this systematic review examined reasons for septoplasty failure. In these studies, missed nasal valve abnormalities, insufficient separation and resection of the bony-cartilaginous junction, and insufficient correction of caudal septal deviation were found to be related to septoplasty failure. Moreover, iatrogenic abnormalities also contributed to failed septoplasty.

In a study published in 2016, Derin et al. [9] reported that anomalies left unresolved following primary septoplasty included deviation of the perpendicular plate of the ethmoid bone, caudal septal deviation, inferior turbinate hypertrophy, concha bullosa, and alar collapse. This study also concluded that following primary septoplasty, iatrogenic surgical deformities and untreated diseases were the main reasons for persistent or recurrent nasal blockage [9].

After the initial septoplasty, the nasal blockage could persist or return [7], requiring revision surgery. Currently, no globally acknowledged septal abnormality categorization system exists for the assessment and identification of the degree of septal deviation. However, nasal septal deviation is a common abnormality causing nasal obstruction [13]. Nasal blockage is also linked to inflammatory conditions, such as allergic rhinitis and nasal polyposis, as well as to structural malformations, such as defects of the nasal structures, including nasal septal deviation and nasal valve issues. To enhance nasal breathing, these abnormalities must be addressed separately [13].

Derin et al. [9] emphasized the significance of a complete physical assessment and objective testing to correctly identify where the nasal blockage is located. Furthermore, to minimize adverse outcomes, attentive postoperative care is essential, in addition to a thorough surgical approach that addresses all diagnosed disorders [9].

In 2018, Jin et al. [10] investigated the factors that led to permanent septal deviation in 100 patients who underwent revision septoplasties. They reported that the middle and caudal septa are common locations for chronic deviation. In addition, insufficient separation and removal of the bony-cartilaginous junction and insufficient restoration of caudal septal deviation were the leading causes of persisting septal deviation after primary septoplasty. Therefore, appropriate chondrectomy, with the removal of the deviated central septal region and batten graft repair of the caudal distortion, is essential for treating chronic deviation [10].

Furthermore, related to recurrent or persistent septal deviation, improper repair of the deformity was reported as a cause of septoplasty failure [14]. In addition, recurring septal deviation due to overlooked nasal valve collapse, turbinate problems, and uncontrolled allergic rhinitis are all associated with the recurrence of nasal blockage after the first septoplasty [14].

The 2008 study by Becker et al. [7] in Virginia included 477 patients who underwent septoplasty and 70 who required corrective septoplasty after their original septoplasty. However, complete surgical records and data for the initial septoplasty were only available for 25 of the 70 patients. When ancillary nasal airway procedures were examined, 93 (19%) of the 477 non-revision patients also underwent nasal valve repair during the original septoplasty. In contrast, only one (4%) of the 25 patients who underwent revision had a nasal valve repaired at the time of the initial septoplasty. Using multivariate analysis, individuals who underwent nasal valve procedures at the time of their initial septoplasty were shown to have a significantly lower chance of needing revision septoplasty than those who did not. Hence, they suggested that patients must be thoroughly assessed before they undergo septoplasty [7].

Similarly, in 2015, Chambers et al. [8] investigated whether patients with a history of unsuccessful septoplasty had a higher prevalence of identifiable structural risk factors. The study also examined changes in the quality of life of patients who underwent nasal valve repair after unsuccessful septoplasty. During the preoperative nasal examination, 38 patients (95%) had internal nasal valve narrowing, 19 (48%) had internal nasal valve collapse, and 16 (40%) had exterior nasal valve narrowing. The authors concluded that surgical nasal valve repair substantially alleviated nasal blockage in patients who underwent failed septoplasty [8].

In a survey of otorhinolaryngology specialists who were members of the Canadian Society of Otolaryngology-Head and Neck Surgery in 2019, Wang et al. [15] found that the most common reason for septoplasty failure in their practice was incomplete septoplasty, followed by untreated nasal valve collapse.

According to Becker et al., the dorsal and caudal septa are the most typical locations for residual deviation [7]. The vast majority of cases in the revision group (48%) had various (caudal and dorsal) locations of septal deviation. A study by Gillman et al. showed that most of the primary septoplasty inadequacies occurred in areas that affect airflow through the internal or external nasal valves. The two most typical sites of residual deviation were the dorsal cartilaginous septum (92%) and anterior (dorsal) bony septum (79%) [5].

As per various scientific papers, crosshatching incisions were not successful in achieving correction due to inadequate extrinsic stresses produced by the nearby bone structures [5,7]. According to other studies, vigorous crosshatching incisions harm the cartilage and result in overcorrection [16,17]. Additionally, straightening the septal cartilage with a crosshatching incision without sufficient battening failed to correct the curvature of the septum or resulted in another deformity, particularly in people of Asian descent with thin and brittle septal cartilage.

To correct the caudal deviation and stop further nasal deformities brought on by the deterioration of the caudal septal support, a batten graft has been developed [18,19]. In revision procedures, in particular, batten grafts are routinely utilized to strengthen the caudal septum that had been weakened and collapsed due to prior resection. The septum can be straightened with adequate caudal septal and dorsal support [18,19].

It should be noted that nasal obstruction is a subjective experience; hence, its cause may not always be apparent. Bohlin and Dahlqvist found that patients who needed revision septoplasty had chronic obstruction [20].

Based on our findings in this systematic review, the leading causes of persistent septal deviation and septoplasty failure are insufficient separation and resection of the bony-cartilaginous junction and inadequate correction of caudal septal deviation [10]. In addition, underdiagnosis of nasal valve dysfunction should be considered in all patients with septal deviation prior to septoplasty [7,8].

Performing septoplasty requires adequate knowledge and prior experience. To accomplish consistent results, surgeons need to comprehensively evaluate the available literature and incorporate the knowledge into their surgical practice. Once the risk factors have been identified, mistakes should be avoided. If there are no indications of mistakes in patient selection, preoperative planning, or operational technique, unsatisfactory outcomes can be attributed to complications [21,22].

Although the present study presents numerous essential and valuable observations, it is limited by the inherent diversity of the methodology. For instance, the study design, the age of the studied patients, and the analysis timing were all characteristics that made it impractical to perform a meta-analysis.

## Conclusions

Identifying the exact reason for nasal obstruction could be challenging, given that this condition is a subjective experience. However, our review of the existing literature revealed that in addition to incomplete separation and resection of the bony-cartilaginous junction, inadequate correction of caudal septal deviation, iatrogenic abnormalities caused by surgery, and ignoring the diagnosis of nasal valve abnormalities and diseases missed during the septoplasty could result in primary septoplasty failure.
